# Is there a trans-abdominal testicular descent during the second gestational trimester? Study in human fetuses between 13 and 23 weeks post conception

**DOI:** 10.1590/S1677-5538.IBJU.2015.0301

**Published:** 2016

**Authors:** Luciano A. Favorito, Fabio O. Bernardo, Suelen F. Costa, Francisco J. B. Sampaio

**Affiliations:** 1 Unidade de Pesquisa Urogenital, Universidade Estadual do Rio de Janeiro, RJ, Brasil

**Keywords:** Cryptorchidism, Gubernaculum testis, Human fetuses, Testicular migration

## Abstract

**Objectives:**

To confirm if a real inner descend of testis occurs, correlating the testicular position with fetal parameters and analyzing the position of the testes relative to the internal ring.

**Material and Methods:**

Twenty nine human fetuses between 13 and 23 weeks post conception (WPC) were studied. The fetuses were carefully dissected with the aid of a stereoscopic lens with 16/25X magnification and testicular position observed. With the aid of a digital pachymeter the distance between the lower pole of the kidney and the upper extremity of the testis (DK-T) was measured to show the position of the testis. During the dissection we also indicated the position of the testes relative to the internal ring. Means were statistically compared using simple linear regression and the paired T-test.

**Results:**

The 58 testes had abdominal position. The DK-T in the right side measured between 0.17 and 1.82cm (mean=0.79cm) and in the left side it was between 0.12 and 1.84cm (mean=0.87cm), without statistically differences (p=0.0557). The linear regression analysis indicated that DK-T in both sides correlated significantly and positively with fetal age. All fetuses with more than 20 WPC, heavier than 350g and with CRL over 22cm had a greater distance than the average DK-T. We xobserved that the 58 testis remains adjacent to the internal ring throughout the period studied.

**Conclusions:**

The testes remains adjacent to the internal ring throughout the period studied, indicating that there is no real trans-abdominal testicular descent during the second gestational trimester.

## INTRODUCTION

Testicular descent is a process that depends on anatomic modifications and hormonal stimulus (1, 2). During the human fetal period, the testes migrate from the abdomen to the scrotum, traversing the abdominal wall and the inguinal canal between the 15^th^ and the 28^th^ week post-conception (WPC) (1-4). Some authors suggest that testicular descent has two separate stages: the first phase corresponds to the testicular descent from the abdomen to the internal inguinal ring, and the second phase corresponds to the transition of the testes through the inguinal canal until their definitive arrival in the scrotum (1-3).

Various factors have been proposed as causing testicular descent in humans, including the increase in the intra-abdominal pressure (5, 6), the development of the epididymis, spermatic vases, deferent ducts and inguinal canal (7); stimuli from the genito-femoral nerve (8); hormonal stimulus originating in the placental gonadotrophin and the testosterone produced by the fetal testes (9); propulsion by the smooth muscle that surrounds the processus vaginalis (10) and gubernaculum development (7).

The moment when testicular descent begins is controversial. Backhouse (2) reports that this pro**cess starts at about the 24** ^**th**^ **week post-conception**, while Heyns (3) and Sampaio&Favorito (4) relate cases where the descent process started as early as the 17^th^ week.

There are only a few reports in the literature about the chronology of testicular descent in human fetuses (11). The process of descent of the testis from the abdomen to the inguinal canal during the first phase of testicular descent is not totally understood in humans. This inner testicular descent was dependent of the gonad growth, involution of mesonephros and the descending septum transversum of the anlage of the diaphragm (12).

The objective of this paper is to confirm if a real inner descend of testis occurs, analyzing the abdominal testicular descent by correlating the testicular position with fetal age, weight, crown-rump length (CRL), total length of the fetus and the position of the testes relative to the internal ring.

## MATERIAL AND METHODS

This study received institutional review committee approval and was carried out in accordance with the ethical standards of the hospital’s institutional committee on human experimentation.

During the period from January 2014 through March 2015, 29 male human fetuses (58 testes) ranging in age from 13 to 23 weeks post-conception (WPC) were studied. The fetuses were macroscopically well preserved, showed no signs of malformations and came to our laboratory as a donation of the obstetric section of our hospital and the demise was hypoxia. The gestational age of the fetuses was determined in WPC, according to the foot-length criterion. This criterion is currently considered the most acceptable parameter to calculate gestational age (13-15). The fetuses were also evaluated regarding total length (TL), crown-rump length (CRL) and body weight immediately before dissection. The same observer made all the measurements.

After the measurements, the fetuses were carefully dissected with the aid of a stereoscopic lens with 16/25X magnification. The abdomen, pelvis and inguinal canal were opened to identify and expose the urogenital organs.

Testicular position was classified after dissection into: a) Abdominal, when the testis was proximal to the internal ring; b) Inguinal, when the testis was found between the internal and external inguinal rings; and c) Scrotal, when the testis had passed beyond the external inguinal ring and was inside the scrotum.

**Figure 1:**
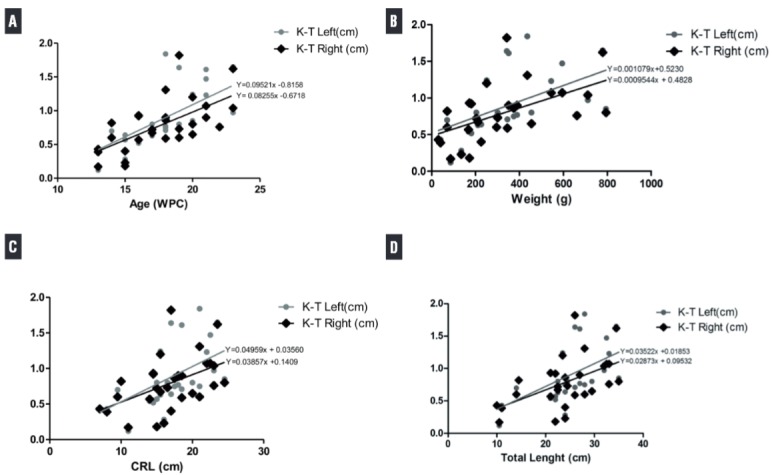
Correlation of the distance between the lower pole of the kidney and the upper extremity of the testis (DK-T) in the left and right side with fetal age, total fetal length, crown-rump length and weight during the fetal period studied (13 to 23 weeks post-conception-WPC). The points plotted represent the mean values obtained for each week studied. A) AGE (WPC). Linear regression indicated that DK-T is correlated significantly and positively with fetal age (right side: r2 = 0.4128, p=0.0002 and left side: r2 = 0.4452, p<0.0001). B) Fetal weight (g). Linear regression indicated that DK-T is correlated significantly and positively with fetal weight (right side: r2 = 0.2987, p=0.0022 and left side: r2 = 0.3094, p=0.0017). C) Crown-rump length (cm). Linear regression indicated that DK-T is correlated significantly and positively with fetal crown-rump length (right side: r2 = 0.2235, p=0.0096 and left side: r2 = 0.2995, p=0.0021). D) Total length (cm). Linear regression indicated that DK-T is correlated significantly and positively with fetal weight (right side: r2 = 0.2729, p=0.0036 and left side: r2 = 0.3324, p=0.0011).

With the aid of a digital pachymeter, the distance between the lower pole of the kidney and the upper pole of the testis (DK-T) was measured to show the descent of the testis during migration. The same observer made these measurements (Figure-1). During the dissection we also indicated the position of the testes relative to the internal ring.

### Statistical analysis

Mean values of the distance between the lower pole of the kidney and the upper extremity of the testis for each side were statistically compared using the unpaired T-test. We also performed simple linear regression to assess the association between the variables analyzed with fetal age. In addition, the correlation coefficient (r) and the p-value were obtained for each regression analysis, and p≤0.05 was considered to indicate statistical significance. The Graph pad Prism 5.0 software was used.

## RESULTS

The fetuses weighed between 30 and 793.4g; had crown-rump length between 7 and 24.5cm and had total length between 10 and 34.5cm. The 58 testes all had abdominal position. The DK-T in the right side measured between 0.17 and 1.82cm (mean=0.79cm; SD=0.3868 and SE=0.07183) and in the left side between 0.12 and 1.84cm (mean=0.87; SD=0.4296 and SE=0.07978), without statistically significant difference (p=0.0557). Table-1 reports the fetal parameters and the measurements of DK-T in the right and in the left side of all fetuses studied.

Considering the average of DK-T in the right and in the left side, all fetuses with more than 20 WPC had a greater distance than the average DK-T value. All fetuses heavier than 350g and with CRL greater than 22cm had the high or very near average DK-T values.

**Figure 2:**
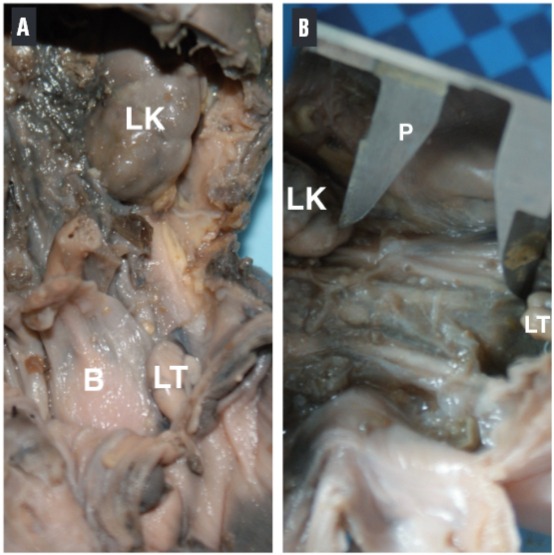
Measurement of the distance between the lower pole of the kidney and the upper extremity of the testis. A) Male fetus with 22 weeks post-conception. The abdominal wall and the intra-peritoneal organs were removed, revealing the left kidney (LK) and the left testis (LT). B=bladder. B) The same fetus, where the distance between the lower pole of the left kidney (LK) and the upper extremity of the left testis (LT) was measured with a digital pachymeter (P).

The linear regression analysis indicated that DK-T in the right and left sides correlated significantly and positively with fetal age and weight, during the fetal period studied (13 to 23 WPC). When comparing the DK-T with TL and CRL, we observed a weak correlation in the right and in the left side. Figure-2 shows the correlations graphs, the linear regression values (r^2^) and the p-values of all fetal parameters studied.

**Figure 3:**
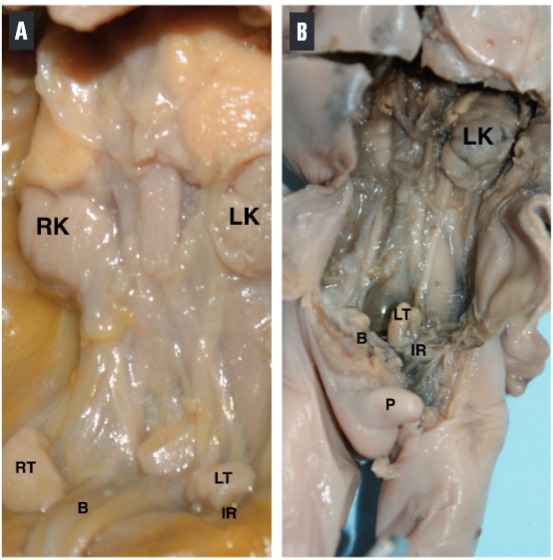
Position of the testes relative to the internal ring. A) Male fetus with 13 weeks post-conception. The abdominal wall and the intra-peritoneal organs were removed, showing that the right (RT) and the left testis (LT) remains adjacent to the internal ring (IR). B) Male fetus with 23 weeks post-conception. The abdominal wall and the intra-peritoneal organs were removed, showing that the right (RT) and the left testis (LT) remains adjacent to the internal ring (IR). LK=left kidney; RK=Right Kidney; B=bladder and P=Penis.

We observed that the 58 testis remained adjacent to the internal ring throughout the period studied (Figure-3).

## DISCUSSION

Previous studies show that in the first phase of testicular descent, the testis descends from the lower pole of the kidney to the pelvic cavity near the bladder neck (16). In this phase, the gubernaculum enlarges to hold the testis near the internal ring, regulated by insulin-like-3-hormone (INLS-3) (17, 18). INSL-3 is secreted by the Leydig cells and controls gubernaculum swelling via its receptor, a process resulting in thickening of the gubernaculum because of increases in water, glycosaminoglycan and hyaluronic acid content (17-21).

The first phase of testicular descent in human fetuses begins around the 8^th^ WPC and lasts until the 15^th^ WPC (22) and the second phase, or the inguino-scrotal phase, begins around the 25^th^ WPC and lasts until the 35^th^ WPC (23). Previous papers studying large numbers of testes show that some fetuses with 17 WPC already had the testis situated in the inguinal canal and all the fetuses older than 30 weeks already had the testes in the scrotum (4). Other authors, however, have reported that the testicular descent is only completed after the 32^nd^ week post-conception (1-3). So the chronology of testicular descent during the second and the third trimesters of gestation are still controversial in the literature.

Previous studies in human fetuses have shown that until 21 WPC, the majority of the testes are located in the abdomen (4, 11). Sampaio&Favorito (4) studied 71 human fetuses (142 testes) and 88% of the testes in fetuses with less than 21 WPC were in the abdomen. Favorito (11) recently studied the asymmetry during testicular migration in 164 human fetuses and observed that 99% of the testes were abdominal in fetuses with less than 20 WPC. These two previous papers only report observations, without any measurements of the testicular descent in the abdomen.

In our sample, formed only of fetuses with 23 WPC or less, all the testes were abdominal, showing that the passage of the testis through the inguinal canal rarely occurs before the 20th WPC. Heyns (3) found only 2.6% of the testes examined in his sample located in the inguinal canal, while Sampaio&Favorito (4), in a sample of 71 human fetuses, found 20.5% of the testes located there. Furthermore, 73.3% of these testes were in fetuses with ages between 21 and 25 WPC, indicating that in this period the migration through the inguinal canal intensifies.

Various parameters have been proposed to determine the gestational age of human fetuses, and crown-rump length and fetal weight are some of the most important (24, 25). Studies correlating fetal parameters with testicular migration during human fetal period are rare in literature. A previous study of fetal weight and testicular descent in human fetuses showed that almost 7% of the testes in fetuses with weight up to 500g had the testes positioned in the inguinal canal (26). In our sample, we observed that the distance between the kidney and the testis was greater in fetuses weighing more than 350g. This information indicated a progressive increase in abdominal length during gestation, and therefore do not convincingly show that the testis has progressive descent during the second trimester.

The only study that correlates the crown-rump length with testicular descent in human fetuses showed that more than 80% of the fetuses with CRL between 6.4 and 20.5cm had testes in the abdominal position and in the fetuses with CRL between 21 and 25.5cm, 45% of the testes were in the abdomen, 45% in the inguinal canal and more than 9% in the scrotum (4). In our sample, all the fetuses had the testes in abdominal position, but we observed that the fetuses with CRL longer than 22cm had high or near average DK-T values, suggesting also a progressive increase in abdominal length during gestation.

In the present study we measured the distance between the lower pole of the kidney and the testis, a very easy way to show the testicular position during the abdominal phase of testicular migration. This measurement can be easily correlated with the fetal parameters too. We observed that the DK-T correlated significantly and positively with fetal age and weight, in the right and left side.

We observed that all the 58 testes of our sample remains adjacent to the internal ring throughout the period studied (13 to 23 WPC), indicating that there is no real trans-abdominal descent of the testis during the second trimester. Previous studies indicated that the testis has progressive descent until the 15^th^ WPC and not during the second gestational trimester, which could be confirmed by our findings (22, 23).

We should mention some limitations of this study: 1) Small sample size–access to human fetuses is limited, so observations of this sample of 29 fetuses may be important although the small number is a weakness, mainly regarding statistical results. 2) Unequal WPC distribution in the period studied–we did not have fetuses with less than 13 WPC and at some ages we had 4 fetuses and at others only 1 or 2 fetuses. Nevertheless, the sample distribution during this important period of testicular migration was adequate in our opinion.

## CONCLUSION

The distance between the kidney and the testis in both sides had a strong positive correlation with fetal age and weight in human fetuses. The testes remains adjacent to the internal ring throughout the period studied (13 to 23 WPC), indicating that there is no real trans-abdominal testicular descent during the second trimester.
